# Rhizoma Coptidis and Berberine as a Natural Drug to Combat Aging and Aging-Related Diseases via Anti-Oxidation and AMPK Activation

**DOI:** 10.14336/AD.2016.0620

**Published:** 2017-12-01

**Authors:** Zhifang Xu, Wei Feng, Qian Shen, Nannan Yu, Kun Yu, Shenjun Wang, Zhigang Chen, Seiji Shioda, Yi Guo

**Affiliations:** ^1^Acu-moxibustion and Tuina Department, Tianjin University of Traditional Chinese Medicine, Tianjin 300193, China; ^2^Acupuncture Research Center, Tianjin University of Traditional Chinese Medicine, Tianjin 300193, China; ^3^South Branch of Guang’anmen Hospital, China Academy of Chinese Medical Science, Beijing 102618, China; ^4^Dongfang hospital, Beijing University of Chinese Medicine, Beijing 100078, China; ^5^Peptide Drug Innovation, Global Research Center for Innovative Life Science, Hoshi University School of Pharmacy and Pharmaceutical Sciences, Shinagawa, Tokyo 142-8501, Japan

**Keywords:** Rhizoma coptidis, berberine, aging, aging-related diseases, AMPK, anti-oxidation

## Abstract

Aging is the greatest risk factor for human diseases, as it results in cellular growth arrest, impaired tissue function and metabolism, ultimately impacting life span. Two different mechanisms are thought to be primary causes of aging. One is cumulative DNA damage induced by a perpetuating cycle of oxidative stress; the other is nutrient-sensing adenosine monophosphate-activated protein kinase (AMPK) and rapamycin (mTOR)/ ribosomal protein S6 (rpS6) pathways. As the main bioactive component of natural Chinese medicine rhizoma coptidis (*RC*), berberine has recently been reported to expand life span in *Drosophila melanogaster,* and attenuate premature cellular senescence. Most components of *RC* including berberine, coptisine, palmatine, and jatrorrhizine have been found to have beneficial effects on hyperlipidemia, hyperglycemia and hypertension aging-related diseases. The mechanism of these effects involves multiple cellular kinase and signaling pathways, including anti-oxidation, activation of AMPK signaling and its downstream targets, including mTOR/rpS6, Sirtuin1/ forkhead box transcription factor O3 (FOXO3), nuclear factor erythroid-2 related factor-2 (Nrf2), nicotinamide adenine dinucleotide (NAD^+^) and nuclear factor-κB (NF-κB) pathways. Most of these mechanisms converge on AMPK regulation on mitochondrial oxidative stress. Therefore, such evidence supports the possibility that rhizoma coptidis, in particular berberine, is a promising anti-aging natural product, and has pharmaceutical potential in combating aging-related diseases via anti-oxidation and AMPK cellular kinase activation.

Aging is a biological phenomenon that is associated with progressive cellular senescence manifesting as growth arrest, impaired function and a decline in metabolism [1-3]. Prior biomedical studies have largely focused on the pathogenesis and treatments of aging-related diseases, rather than the relationship between aging and disease on a molecular level, as it was thought that to be able to directly target aging was more or less theoretical. People are thus living longer, but continue to suffer the disabilities and morbidities associated with aging and aging-related diseases [4]. The focus is now shifting as multiple studies have identified several key mechanisms of aging, and targeted interventions that modified those mechanisms, which have translated into clinical applications, such as dietary restriction, exercise, rapamycin (mTOR) inhibitors, adenosine monophosphate-activated protein kinase (AMPK) activators, nicotinamide adenine dinucleotide (NAD^+^) precursors, sirtuin1 (SIRT1) activators, modifiers of senescence and telomere dysfunction, hormonal and circulating factors including sex-steroids and growth hormones, and mitochondrial targeted antioxidants [5].

Cumulative DNA damage caused by reactive oxygen species (ROS) in mitochondria has long been considered as the primary cause of aging [6]. According to Harman's mitochondrial free radical theory of aging [7], oxidative stress within the organelle leads to accumulation of mitochondrial DNA (mtDNA) mutations, increased superoxide production, and a vicious cycle of oxidative stress. This further accelerates mtDNA mutagenesis and worsens mitochondrial function [8]. Aging-associated failures in mitochondrial maintenance, such as reduced mitochondrial autophagy rate (mitophagy) is controlled by the AMPK, mTOR, and forkhead box transcription factor O3 (FOXO3) signaling pathways [9, 10]. Meanwhile, mitochondrial malfunction is a key underlying factor in inducing mis-regulation of apoptosis, chronic inflammation and premature senescence during aging [8, 11,12]. In fact, mtDNA mutant mice expressing a proofreading-deficient version of mitochondrial DNA polymerase g (POLG) accumulated mtDNA mutations and displayed features such as gray hair, thin skin, osteoporosis, anemia, premature cease in fecundity and a shortened life span, all signs associated with advancing age [13, 14].

Chinese herb rhizoma coptidis (*RC*) has historically been used over the past 3000 years for its potent anti-diarrheal and anti-microbial effects, particularly against Chlamydia and protozoans [15, 16]. *RC* and its main bioactive components have attracted attention in recent years owing to its wide spectrum of pharmacological effects, particularly in aging-related diseases, including that against hypertension, hyperglycemia, hyperlipidemia, cancer, arrhythmia, depression, etc [17, 18]. Key pathologies of aging, including oxidative stress, mitochondrial malfunction, and the associated mTOR, AMPK and NAD^+^ signaling pathways have been verified to be regulated by *RC* components, especially berberine [4, 19, 20]. Furthermore, *RC* can directly recognize and bind nucleic acids and various proteins, including telomerase, DNA topoisomerase, p53, NF-κB, MMPs and estrogen receptors, thereby can potentially alter aging-associated cellular processes [21-23].

We thus propose that *RC* can prevent aging and treataging-associated disorders by targeting multiple aging-associated signaling pathways. This review attempts to summarize the underlying mechanisms and pathways *RC*’s multi-spectrum anti-aging activity.

## 1. *RC’s* main constitutents

### 1.1 Introduction of *RC* and its main components

**Figure 1.**
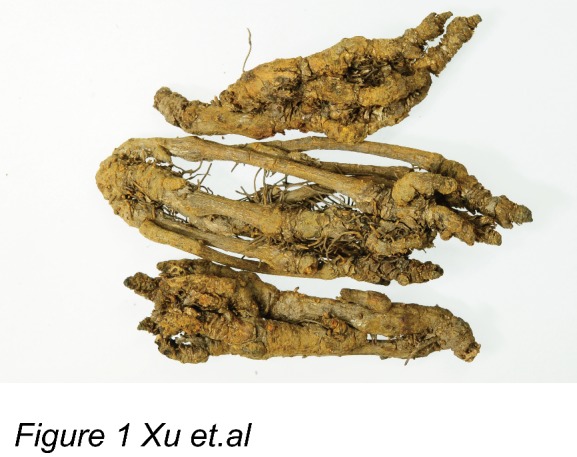
The photographs of *RC* dried root and rhizome.

*RC* is the dried root and rhizome of three *Coptis* species: *Coptis chinensis* Franch, *Coptis deltoidea* C. Y. Cheng et Hsiao., and *Coptis teetoides* C. Y. Cheng (Fig. 1). There are a few other native *Coptis* species distributed in other regions, i.e. *Coptis trifolia* Salisb*,* and *Coptis japonica* Makino. In China, *RC* has been extensively cultivated in eastern Sichuan and western Hubei provinces under good agriculture practice (GAP) for crude drugs. It is known that the *Coptis deltoidea* C. Y. Cheng has been considered as vulnerable with the conservation status ranking No. 2 in China [24, 25].

**Figure 2.**
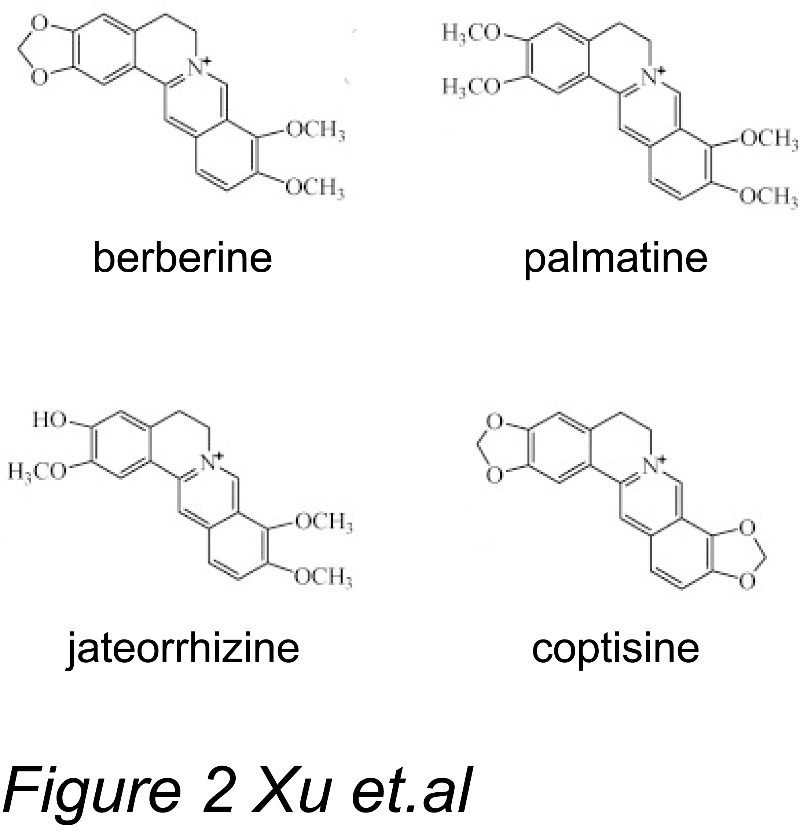
The structures of berberine and other key protoberberine-type alkaloids contained in *RC*.

*RC* is composed of diverse alkaloids, including berberine (6.88% to 13.64%), palmatine (1.28% to 2.12%), jatrorrhizine (0.77% to 1.32%), coptisine (0.42% to 0.85%), and epiberberine (0.42% to 0.92%), with berberine being the primary compound. The structures of berberine and other key protoberberine-type alkaloids contained in *RC* are shown in Figure 2 [26]. The purification of berberine from *RC* has significantly facilitated investigational studies into the therapeutic applications of *RC*. The structure of berberine represents a biologically important skeleton, and a natural lead compound that can be chemically modified (Fig. 2). Berberine was firstly isolated and identified as a plant isoquinoline alkaloid in the early nineteenth century. In the years from 1910s to 1960s, the chemistry and synthesis of berberine was thoroughly studied. In the early 1960s, berberine and its salts, such as berberine sulphate, was demonstrated to be valuable to treat infectious diarrhea and amoebiasis by Indian researchers [27]. Berberine was first synthesized in 1969. Berberine’s anti-microbial activity is not inherent, but rather potentiated by the MDR inhibitor, 5′-methoxyhydnocarpin [28]. This study demonstrated that *RC* extract may have more therapeutic effects than berberine alone, which may be due to the synergistic actions of other components in *RC*.

### 1.2 Traditional usages of *RC* and the development of modern pharmacological investigation

Since its first record in Shennong's Materia Medica during the eastern Han dynasty (25-220 AD), *RC* has been widely used for various illnesses by Chinese herbalists for over 2000 years [29]. Since the 1980’s, berberine has been increasingly known worldwide for its wide spectrum of pharmacological effects, particularly in aging-related diseases, including hypertension, hyperglycemia, hyperlipidemia, cancer, arrhythmia, depression, etc. Recently, several studies have also demonstrated the role of *RC* in anti-aging.

### 1.3 Pharmacokinetic profile

Generally, blood concentrations of *RC* alkaloids are extremely low after oral administration, but can be higher in some pathological conditions. The alkaloids exert systemic effects through generated metabolites or by directly modulating effectors in the gut [31]. The pharmacokinetic profile of berberine and its metabolites has been well studied in humans and animals [19, 32]. Berberine is present at a very low level in blood due to its poor aqueous solubility and dissolution [33], but the pharmacological effect of berberine is correlated with its high tissue distribution. It has been reported that berberine can also enter the blood-brain barrier, and is slowly eliminated from the CSF, and it can be highlighting its neuroprotective role [34]. Specifically, berberine is more rapidly cleared from plasma compared to hippocampus, indicating that berberine could have a direct action on hippocampal neurons [35]. In order to improve the bioavailability of berberine, D-a-Tocopherol polyethylene glycol 1000 succinate and P-glycoprotein have been demonstrated to increase its intestinal absorption. Several formulations have been devised to improve its bioavailability by a noral berberine-loaded micro-emulsion [36]. In addition, an anhydrous reverse micelle system of berberine prepared via lyophilization of water-in-oil emulsions was also reported to show enhanced anti-diabetic efficacy attributed to higher bioavailability [37]. Wang *et al.* examined berberine’s after intravenous administration in rats: half-life: 0.22 h, maximum thalamus concentration: 272 ng/g, time to peak concentration: 3.67 h and elimination half-life:12.0 h [35].

## 2. Anti-aging effects of *RC* component berberine

### 2.1 Prolongation of life span and health

*Drosophila melanogaster* is a valuable model for preclinical testing of drugs with therapeutic potential for aging, and aging-associated medical and psychiatric disorders (AAMPD) because of its small genomic size, short generation time, and short mean life span compared to both mice and humans with similar vulnerability to age-relate decline. Activation of the kynurenine pathway of tryptophan metabolism is an important contributor to AAMPD, since genetic deficiency and pharmacologic inhibition of kynurenine formation from tryptophan in *Drosophila* was shown to prolong life span [38] and have a neuroprotective effect in flies [39]. Recent studies have found that the major component of *RC*, berberine, could extend the life span of female *Drosophila melanogaster* by inhibiting the enzyme that catalyzes tryptophan conversion into kynurenine. While RC may prolong life span, it also has benefits on overall health. Berberine was demonstrated to stimulate locomotor activity in *Drosophila,* is a crucial biomarker for the flies’ health [38]. It is known that life span is temperature-dependent, and flies were dying more quickly at a higher temperature. The same research group demonstrated involvement of tryptophan-kynurenine metabolism in the aging-acceleration effect of elevated temperatures, as well as in protective effect of berberine [40]. Despite this, the role of berberine in the prolongation of mammal life span has not been reported [40].

Berberine has also been found to specifically have an anti-aging effect on skin. First, berberine prevented TPA-induced ERK activation and AP-1 DNA binding activity, which can prevent skin inflammation and degradation of extracellular matrix proteins [41]. Second, berberine decreases both basal and UV-induced MMP-1 expression, but increase type I procollagen expression in human dermal fibroblasts [42].

### 2.2 Attenuation of premature cellular senescence

Cellular senescence is a process that imposes permanent proliferative arrest on cells in response to repeated cell divisions and telomere erosion, as well as to activated oncogenes, disrupted chromatin structure and oxidative stress [43]. Senescent cells are resistant to apoptosis [44], which may be why they have been observed to accumulate during aging in several tissues [45]. A growing body of evidence suggests that the drawbacks of senescence are twofold. First, senescence causes a loss of tissue-repair capacity because of cell cycle arrest in progenitor cells. Second, senescent cells produce pro-inflammatory cytokines and extracellular matrix degrading enzymes [46].

Cellular senescence can be categorized into two types. The replicative senescence is featured as the loss of replicative capacity of normal diploid cells after a certain number of cell divisions (Hayflick’s limit). Erosion of telomeres at each cell division resulting in telomere dysfunction is the primary cause of irreversible cell cycle arrest defined either as intrinsic or replicative cellular senescence. The second category is premature senescence, which is independent of telomere shortening. Among the factors inducing premature senescence are persistent DNA replication stress, oncogenes activation, and loss of tumor suppressor genes [47]. The stress-induced premature senescence of normal cells *in vivo* is considered to be a critical mechanism affecting aging and longevity of the organism [48].

Administration of berberine into cultures of A549 cells undergoing premature senescence reduced the development of senescent phenotype, evidenced by as analysis of cells morphometric features through laser scanning cytometry. The increase in the ratio of mean intensity of maximal pixels to nuclear area is considered to be a very sensitive morphometric biomarker for the degree of senescence (the decline in local intensity of DNA-associated DAPI fluorescence represented by maximal pixels is paralleled by an increase in nuclear area) [49, 50]. The characteristic features of cellular morphology combined with immunocytochemical detection of senescence markers, such as overexpression of cyclin kinase inhibitors (e.g., p21WAF1) and phosphorylation of rpS6 are considered to be the most specific biomarkers of senescent cells [51]. Another attribute of senescent cells is persistent expression of markers of DNA damage such as γH2AX and activation of ATM, a consequence of oxidative DNA damage by endogenous oxidants [52]. These biomarker indices are presented in quantitative terms defined as the senescence index (SI), which is the fraction of the marker in test cultures relative to the same marker in exponentially growing control cultures. By using this system, administration of berberine also activated SA-β-gal and induced CDK inhibitor p21WAF1, p16INK4A, and p27KIP1, and through activation of tumor suppressor p53 signaling pathways, attenuated the level of mTOR/rpS6 signaling by lowering the level of phosphorylation of rpS6 and expression of γH2AX [47, 50].

## 3 Actions of *RC* and its key components in aging related diseases

**Table 1:** Anti-aging effect of bererine.

Refs.	Cell type, animal model	Effect of BBR	Index
[37]	wild-type Drosophila melanogaster	boosts life-span and health-span	mean, median and maximum life span, locomotor activity (vertical climbing), lethality, kynurenine, tryptophan
[39]	wild-type Drosophila melanogaster	boosts life-span	kynurenine, tryptophan
[49]	premature senescence in pulmonary adenocarcinoma A549 cells induced by persistent DNA replication stress	anti-senescence	cell morphology; the ratio of mean intensity of maximal pixels to nuclear area; cyclin kinase inhibitors, phosphorylation of ribosomal protein S6 (rpS6)
[41]	normal human keratinocytes	anti-skin aging	TPA, MMP-9, IL-6, ERK, AP-1 DNA binding activity
[42]	human dermal fibroblasts	anti-skin aging	MMP-1, type I procollagen
[83]	hydrogen peroxide-induced senescent cells	anti-oxidation, promote autophagy,	p62, NAD^(+)^, mTOR, Sirt1

Besides anti-aging actions shown in Table 1, *RC* and its key components have been demonstrated to have a broad array of beneficial pharmacological effects on aging-related diseases, including hyperlipidemia, hyperglycema, hypertension, arrhythmias, neuro-degeneration, depression, anxiety, etc (Table 2) [17, 18].

### 3.1 Hypolipidemic activity

The hypolipidemic actions of berberine have been confirmed in humans. In 2004, Kong *et al.* found that oral administration of berberine in patients with hypercholesterolemia for 3 months could reduce total cholesterol by 29%, triglycerides by 35%, and low density cholesterol (LDL) by 25% and that berberine up-regulates LDLR expression dependent on ERK activation [53]. Zhou *et al.* showed that berberine modulated glycolipid metabolism through increasing peroxisome proliferator-activated receptors (PPARs) PPAR*α*/*δ* expression and reducing PPAR*γ* expression in liver. *In vitro* data showed that coptisine could up-regulate mRNA and protein expressions of LDLR and cholesterol 7*α*-hydroxylase (CYP7A1), and down-regulate that of HMGCR, so as to reduce total cholesterol and LDL levels [54]. In hamsters on a high fat diet, coptisine significantly decreased the levels of blood lipid [55]. Jatrorrhizine-treated hyperlipidemic mice exhibited reduced serum triglyceride, total cholesterol and low LDL levels, as well as increased HDL levels compared with untreated mice [56]. Wu *et al.* showed that jatrorrhizine had a strong dose-dependent hypolipidemic effect mainly through up-regulation of mRNA and protein expression of LDLR and CYP7A1 [26, 57]. Palmatine was also found to show anti-hyperglycemic effects by up-regulating LDLR and CYP7A1 mRNA and protein expression, down-regulating sodium-dependent bile salt transporter mRNA and protein expression, as well as enhancing fecal excretion of cholesterol and total bile acids [58].

### 3.2 Anti-hyperglycemic activity

The emerging role of berberine in diabetes mellitus (DM) has been demonstrated in clinical and experimental studies. In general, berberine is safe and effective in the treatment of patients with T2DM by reducing blood glucose, similarly to metformin and rosiglitazone [59, 60]. Notably, complications of diabetes including nephropathy, endothelial dysfunction, and neuropathy also improved with berberine treatment [61]. Berberine may exert its anti-hyperglycemic effects by enhancing insulin sensitivity and increasing insulin secretion [60]. Moreover, Xie *et al.* showed that berberine significantly reduced the number of harmful microbiota and increased the number of beneficial ones in the feces of high-fat diet fed (HFD) mice [62]. In addition, a palmatine-derivative, 11-hydroxypalmatine, could decrease blood glucose in alloxan-induced diabetic mice [63]. Yang *et al* found jatrorrhizine-treated hyperlipidemic mice exhibited a reduction in body weight, as well as improved glucose tolerance and insulin sensitivity [64].

### 3.3 Anti-hypertensive activity

The anti-hypertensive and vasodilator effectsof berberine has been observed in several hypertensive animal models. Berberine has been shown to delay not only the onset, but also attenuated the severity of hypertension, and possible secondary renal damage in spontaneously hypertensive rats (SHR) [65]. Berberine can act on both endothelium and underlying vascular smooth muscle to induce vaso-relaxation via multiple cellular mechanisms, which can involve direct release of NO/cGMP from aortic rings, increased sensitivity to acetylcholine, activation of K^+^ channels and α1-adrenoreceptor-blocking activity [67]. Lower concentrations berberine-mediated aortic relaxation appears to be dependent on its effect on endothelium [66].

**Table 2:** Effect of *RC* major components (bererine, coptisine, jatrorrhizine and palmatine) on aging-related disease.

Component	Refs.	Patient, cell type, animal model	Index
anti-hyperlipidemia
Berberine	[52]	hyperlipidemic patients	TC, TG, LDL-c, LDLR
	[53]	diabetic hyperlipidemic rat	TC, TG, LDL-c, apolipoprotein B, apolipoprotein AI
Coptisine	[54]	HepG2 cells	TC, TG, LDL-c, HDL-c, LDLR, HMGCR, CYP7A1
	[55]	HFHC-induced hyperlipidemic hamsters	body weight gain, TC, TG, LDL-c, HDL-c, TBA
Jatrorrhizine	[55]	hyperlipidemic mice	TC, TG, LDL-c, HDL-c, SREBP-1c, FAS, PPAR-α, CPT1A
	[56]	HFHC-induced hyperlipidemic hamsters	TC, TG, and LDL-c, HDL-c, TBA,LDLR, CYP7A1, HMGR, ASBT
	[57]	high-fat and high-cholesterol (HFHC) mice	TC, TG, LDL-c,
anti-hyperglycemia
Berberine	[59]	type 2 diabetes mellitus patients	blood glucose, insulin receptor
Palmatine	[62]	alloxan-induced diabetic mice	blood glucose
Jatrorrhizine	[63]	high-fat diet-induced obesity and hyperglycemic mice	body weight, blood glucose, insulin receptor
anti-hypertensive activity
Berberine	[64]	spontaneously hypertensive rats	Blood pressure, IL-6, IL-17 and IL-23
Alzheimer’ disease
Berberine	[68]	human neuroglioma H4 cells	APP
Coptisine	[44]	AβPP/PS1 transgenic mice	cognition, neuron loss, amyloid plaque formation, IDO

### 3.4 Combating Alzheimer’ disease (AD)

AD is a devastating neurodegenerative disease affecting the aging population [68]. Berberine is a promising agent to combat AD via its inhibitory effects against oxidants, acetylcholinesterase, butyrylcholinesterase, and mono-amine oxidase, as well as reduction of amyloid-β peptide and cholesterol [69]. Interestingly, berberine can reduce Aβ levels by altering APP processing in human neuroglioma H4 cells that stably express APP without cellular toxicity [70].

The pathogenesis of AD is linked to a deficiency in brain acetylcholine. It has been reported that berberine can inhibit both cholinesterases (ChEs) and beta-amyloids formation, as well as marked ONOO^(-)^ scavenging and ROS inhibitory capacities [71, 72]. Berberine has been demonstrated to inhibit monoamine oxidase MAO-A and MAO-B, both of which accelerates neuro-degeneration in AD models. Meanwhile, Yu *et al.* demonstrated that coptisine significantly inhibited recombinant human IDO activity, shedding light on the mechanism of coptisine’s action on AD [73].

Through the aforementioned effects, *RC* and its components can potentially combate several age-related diseases; however, berberine is the only compound with direct anti-aging effect.

## 4 The potential mechanisms of *RC*/berberine in combating aging and aging-related diseases involving targeting of AMPK signaling

More recently, the persistent stimulation of the mitogen and nutrient sensing pathways including AMPK and TOR/rpS6 signaling has been demonstrated as an alternative to the ROS mechanism in aging process [74, 75]. On one hand, AMPK activation has been found to boost overall health and protects cells from oxidative stress-induced senescence via autophagic ?ux restoration and intracellular NAD^+^ elevation. On the other hand, activation of mTOR pathway enhances translation and leads to cell growth resulting in cell hypertrophy and senescence [76]. Similar to metformin, emerging evidence shows that berberine could be another AMPK activator that regulate both ROS and mTOR/rpS6 pathways, and thereby have the potential to enhance health [77]. AMPK activator metformin and berberine are reported to extend life span of C. elegans [78, 79] and even rodents [80, 81], as mentioned in above.

### 4.1 Structure and regulation of AMPK signaling

AMPK has been considered as the “energy sensor” of the cell, as it is activated by an elevation in the cellular ratio of AMP+ADP to ATP [82]. Mammalian AMPK exists as heterotrimeric complexes that is comprised of a catalytic α subunit and regulatory β and γ subunits. Seven distinct genes code for the various isoforms of AMPK, two for α subunits, two for β, and three for γ. The α subunit has a kinase domain at the N-terminus that is active only after phosphorylation in the activation loop at Thr-172, which is now widely used as a biomarker for AMPK activation [82]. The γ subunit has a regulatory function that contains four tandem repeats of a cystathionine-β-synthase (CBS) sequence numbered 1-4. CBS site 2 appears to remain empty, whereas site 4 constitutively binds AMP. The γ subunit's regulatory function is exerted through CBS sites 1 and 3, each of which can reversibly bind either AMP, ADP, or ATP. AMP binding to site 1 allosterically boosts the kinase activity of the activated enzyme. At site 3, binding of either AMP or ADP suppresses the ability of phosphatases to remove the phosphate from Thr172 and thereby deactivate the enzyme [82].

There are several activating upstream kinases targeting AMPK, including liver kinase B1 (LKB1) and calmodulin-dependent kinase kinases (CaMKK). Although LKB1 also acts upstream of a small family of AMPK-related kinases, AMPK is its only target known to inhibit cell growth and proliferation. AMPK is acutely activated when the (AMP+ADP)/ATP ratio increases, or in response to a rise in cytosol Ca^2+^ condition which often senses cellular stress. For example, HDL particles could lead to an increase in calcium influx that activates CaMKK, and stimulates AMPK signaling in endothelial cells [83]. In addition, the modulation of the phosphatases that target Thr172 of the α subunit-PP2A and PP2C can influence AMPK activity as well [84].

A number of drugs, phytochemicals and hormones have the potential to activate AMPK. For instance, metformin boosts (AMP+ADP)/ATP ratio by impeding the efficiency of mitochondrial electron transport. AMPK can also be activated allosterically by certain agents that bind to it at a site distinct from its AMP/ADP binding region. The ability of the hormone adiponectin to activate AMPK has recently been traced to the ceramidase activity of the activated adiponectin receptor -ceramide suppresses AMPK activity by activating PP2A [84].

### 4.2 Potential mechanisms by which *RC*/berberine activates AMPK signaling

Berberine has long been considered as a AMPK activator by activating AMPK in several cell types, such as endothelium, smooth muscle, cardiomyocytes, cancer cells, β-cell, hepatocytes, macrophages, and adipocytes [85]. Pharmacological activation of AMPK by berberine has also been found to prevent the development of senescence and characterized in the treatment of metabolic and neurodegenerative, as well as and other aging-related diseases [86, 87]. In addition, both *RC* and berberine significantly increase mRNA expressions of AMPK in visceral adipose tissues and livers of high-fat diet-fed mice [62]. The mechanism by which berberine exerts AMPK activation effects may be via mitochondrial targeting. The localization of berberine in mitochondria is photolabile; even short exposure to UV light can result in loss of its mitochondrial localization and translocation into nuclei [88]. The specific target appears to be the respiratory electron transport chain; inhibition of the electron transport results in a decline in content of ATP, an increase of AMP/ATP ratio and triggering of AMPK activation [89, 90]. In addition, berberine significantly increased AMPK activity via ROS production [91].

### 4.3 Downstream targets of the AMPK signaling regulated by berberine

#### Mitochondria

Since AMPK senses cellular energy deficit, it could enhance the capacity of cells to generate ATP via substrate oxidation while simultaneously suppressing the activity of metabolic pathways that utilize ATP [92]. This mechanism is essentially identical to that induced by metformin, which also targets electron transport in complex 1 of mitochondria and thereby activates AMPK [93]. Hence, AMPK boosts mitochondrial biogenesis, mitochondrial antioxidant protection, and increases the expression and activity of glucose transporters and glycolytic enzymes; concurrently, non-essential synthesis of proteins, lipids, and carbohydrates is decreased [92]. Thus, berberine, as a AMPK activator, can combat the aging process by targeting mitochondria. Uncoupling protein 2 (UCP2) is a member of the mitochondrial inner membrane proteins that is negatively related to ROS production and oxidative stress [94, 95]. It has been shown that berberine can enhance UCP2 expression, which in turn inhibits oxidative stress induced atherosclerosis in an AMPK-dependent manner in mice.

#### mTOR signaling

Much evidence has shown that constitutive activation of mitogen and nutrient sensing signaling pathways enhances translation, leads to cell growth in size and mass and ultimately leads to cell hypertrophy and senescence [96-98]. Activation of mTOR when combined with inhibition of cell cycle progression or ongoing oxidative DNA damage has been shown to be the driving force leading to aging and senescence, both at the cellular and organismal level [90, 91]. The most convincing evidence for the mechanism involving mTOR stems from the recent results that show increased life span and improved health of several organisms, including mammals treated with direct (rapamycin) or indirect (e.g., metformin) mTOR inhibitors (rapalogs) [99, 100]. In addition, mTOR has the potential to regulate both resting oxygen consumption and oxidative capacity in mitochondrial activity. The inhibition of mTOR/rpS6 signaling is one of the key effects of AMPK activation [91, 101, 102]. AMPK mimics the impact of growth factor down-regulation associated with calorie restriction by inhibiting activity of the mammalian target of rapamycin complex 1 (mTORC1) [103]. mTORC1 plays a significant role by targeting the process of autophagy and protein synthesis via phosphorylation of its targets p70 rpS6K1 and 4EBP1 [101, 102, 104].

It has been recently reported that berberine could suppress mTOR signaling as evidenced by the reduced level of constitutive phosphorylation of rpS6 on Ser235/236, the key effector of the mTOR/rpS6 signaling, as measured in individual cells by flow and laser scanning cytometry [105]. AMPK/mTOR is the essential regulator in cellular autophagy. The mammalian target of mTOR kinase is a central inhibitor of autophagy. mTORC1 is rapamycin sensitive and acts as a major checkpoint between cell growth and autophagy [102,106]. Rapamycin, a mTOR inhibitor, suppressed the amount of LC3 II/I which was down-regulated by beberine in the presence of rapamycin. Conversely, leucine enhanced the level of LC3 II/I, which was also down-regulated after exposure of berberine. Furthermore, the pretreatment with compound C (AMPK inhibitor) increased the expression of p-mTOR/mTOR, and inhibited LC3 expression and p62/SQSTM1 degradation in oxLDL-stimulated J774A.1 cells (monocyte/macrophage cell line), suggesting that AMPK might be an upstream factor of mTOR, and berberine-induced autophagy may be preceded by the activation of AMPK/mTOR in J774A.1 cells. This further demonstrates that the AMPK/mTOR signaling pathway may be involved in autophagy induced by berberine in macrophages [107].

#### SIRT1/FOXO pathway

Sirtuin 1 (SIRT1) has been considered as an evolutionarily conserved enzyme that mediates the life-prolonging impact of calorie restriction in lower eukaryotes. In light of recent reports that the wine phytochemical resveratrol may exert a pro-longevity effect by activating SIRT1, it should be noted that this activation appears to be indirect, mediated via resveratrol's impact on AMPK [108, 109]. Thereby, AMPK might combat aging via enhancing the activity of SIRT1. FOXO transcription factors have important roles in metabolism, cellular proliferation, stress tolerance, antioxidation and aging. AMPK can also phosphorylate and thereby boost the transcriptional activity of FOXO3a, which induces the expression of a number of antioxidant enzymes and other stress resistance proteins [110]. Since autophagy rids the cell of aging, including potentially clear pro-oxidative mitochondria, it has been found that AMPK, Sirt1, and FOXO3a also collaborate in promoting mitochondrial biogenesis via enhancing autophagy, largely by boosting the expression and pro-transcriptional activity of PPAR-γcoactivator-1α [108, 111]

As an AMPK activator, berberine has been reported to increase the expression level of SIRT1 in the hepatic cell line L02 [112, 113]. In oxidative stress, SIRT1 could induce deacetylation of FOXOs transcription factors and increase FOXO-dependent transcription of stress-regulating genes, such as *SOD* [114]. It is possible that berberine increases SOD expression *via* the AMPK induced SIRT1/FOXO pathway. Berberine also increases the expression of SIRT1 at both the protein and mRNA levels to regulate autophagy in macrophages in a dose-dependent manner [107, 115].

#### Erythroid-2-related factor-2 (Nrf2) pathway

The nuclear factor Nrf2 is an antioxidant transcription factor that mediates the expression of antioxidant enzymes inculding SOD, GSH, heme oxygenase-1 (HO-1) and NADPH quinine oxidoreductase-1 (NQO-1) [116]. The Nrf2 pathway has been found to play an important role in carrying out berberine’s reduction of oxidative stress [117-120]. It has been demonstrated that the antioxidant activity of berberine can be diminished if Nrf2 were to be blocked in macrophages and nerve cells [117, 119]. First, berberine can induce Nrf2 nuclear translocation by activating several cellular signaling pathways, including AMPK [118]. Second, following nuclear translocation, Nfr2 promotes the transcription and expression of several antioxidant enzymes, increase SOD, HO-1 and GSH contents in cells and reduce ROS production and oxidative stress, whereas blocking these pathways could abolish the stimulatory action of berberine on Nrf2 [117-120].

#### Nicotinamide adenine dinucleotide (NAD^+^) pathway

NAD^+^ in cells is another feature of aged organisms [121]. Supplementation with NAD^+^ precursors has been shown to ameliorate or reverse the effects of aging in old worms or mice [122]. Interestingly, AMPK activation raises intracellular NAD^+^ concentrations and activates SIRT1 [115], which is mediated via an increase in theactivity and abundance of NAMPT, a key enzyme in the salvage pathway of NAD^+^ synthesis [123]. Berberine is able to down-regulate the expression of nicotinamide adenine dinucleotide phosphate (NADPH) oxidase in a AMPK-reliant pathway, a major source of ROS production in cells [124, 125]. NADPH oxidase could be up-regulated by high levels of fatty acids, glucose or advanced glycation end products, resulting in ROS overproduction [126]. Among multiple NADPH oxidase isoforms, berberine has been demonstrated to suppress the over-expression of NADPH oxidase 2/4 and decrease ROS production in macrophages and endothelial cells upon stimulation with inflammatory stimuli [124, 125]. In addition, AMPK activation restored NAD^+^ levels in senescent cells via the inhibition of mTOR and Sirt1 activation that is part of the salvage pathway for NAD^+^ synthesis [87]. In addition, AMPK increases the expression of nicotinamide phosphoribosyl transferase, which is rate-limiting for the regeneration of SIRT1’s obligate cofactor, NAD^+^ [108].

#### NF-κB pathway

Aging-associated excessive apoptosis and decreased phagocytosis may contribute to chronic inflammation, including activation of the NF-κB signaling pathway [127], featured as increased circulating levels of pro-inflammatory cytokines (IL-1β, IL-6, TNF-α), acute phase proteins including C-reactive protein and serum amyloid A, ultimately leading to increased frequency of chronic inflammatory diseases of aging such as AD, Parkinson's disease, amyotrophic lateral sclerosis, atherosclerosis, etc [127]. Moreover, there is a significant cell loss of cells in the thymus and bone marrow, which is associated with an age-related increase in the number of apoptotic CD4^+^ and CD8^+^ T cells [128].

Many studies have shown AMPK activation down-regulates NF-κB activation in various cells [129]. It seems that AMPK suppresses NF-κB signaling indirectly via its downstream mediators, e.g., SIRT1, FOXO family, and peroxisome proliferator-activated receptor γ co-activator 1α, which can subsequently repress the expression of inflammatory factors. For instance, it was demonstrated that the knockdown of α1AMPK abolished the anti-inflammatory effect of metformin [130]. Yang *et al.* revealed that the constitutively active α1AMPK suppressed NF-κB signaling and fatty acid-induced inflammation in macrophages and that dominant-negative α1AMPK could reverse the inhibition [131]. Katerelos *et al.* have observed that overexpression of α1AMPK reduced NF-κB signaling in aortic endothelial cells [132]. Wang *et al.* [133] demonstrated that NF-κB signaling was activated in aortic endothelial cells isolated from AMPKα2 knockout mice, whereas AMPK activation by AICAR and constitutively active AMPKα2 had the opposite effect [133].

The primary mechanism of berberine’s anti-inflammatory activity may involve AMPK inhibition of NF-κB dependent pathway [22]. As a transcription factor, NF-??B promotes the expression of various pro-inflammatory cytokines such as TNF-α, IL-6, iNOS and COX2 [134]; it is a critical target for the anti-inflammatory activity of berberine, as well as inhibitory ??B-α (I??B-α) was phosphorylated by IKK-β and then degraded, inhibition of IKK-β by berberine could result in the stabilization of I??B-α [135, 136], which in turn blocked the nuclear translocation of NF-??B [136]. In a variety of cells or tissues like nerve cells, lung cells, pancreatic β-cells, and rat kidney as well as in a mice model of insulin resistance [137], and the inhibitory effect of berberine on the production of pro-inflammatory cytokines was associated with its inhibition of the NF-??B signaling pathway.

## 5. *RC*/berberine’s anti-oxidant activity is a critical component of its anti-aging effect.

### 5.1 The role of the ROS system in the aging process

It is generally believed that persistent DNA damage by ROS generated in mitochondria during oxidative phosphorylation a primary mechanism of aging [6]. DNA double-strand breaks (DSBs), the most deleterious lesions induced by ROS, are repaired either by homologous recombination or non-homologous DNA-end joining (NHEJ). Repair by recombination requires a DNA template and therefore, can take place in cells that already have it replicated. Lesions involving telomeric DNA can lead to a dysfunction of telomeres, thereby impeding DNA repair and driving cells to undergo replicative senescence [138, 139]. Accumulation of unrepaired or incorrectly repaired DNA lesions lowers genome integrity, leading to loss of fidelity of transcription and generation of defective proteins. Meanwhile, aging-dependent oxidative modification of voltage-gated potassium (K^+^) channels, initially demonstrated in the mammalian brain, contributes to altered excitability, progression of normal aging and neurodegenerative disease [140]. In addition, as a precursor of many free radicals, superoxide has been verified to regulate major epigenetic processes of DNA methylation, histone methylation, and histone acetylation, all of which can contribute to aging process [141].

### 5.2 Antioxidant activity of berberine and its components in aging and aging-related diseases

The antioxidant activity of berberine has been widely demonstrated in aging cells *in vitro* and in aging models [87, 137, 139]. Berberine has been found to decrease the level of constitutive DNA damage signaling as seen by the reduced expression of γH2AX in proliferating A549, TK6, WI-38 cells and in mitogenically stimulated human lymphocytes, as well as reduce intracellular ROS level and mitochondrial trans-membrane potential ΔΨm [139]. By using H_2_O_2_-induced senescence model, berberine significantly prevented the development of senescence via activation of AMPK pathway, which prevented hydrogen peroxide-induced impairment of the autophagic flux in senescent cells, and restored NAD^+^ levels in the senescent cells via a salvage pathway for NAD^+^ synthesis [87].

Berberine quenches ROS and reactive nitrogen species (RNS) by exerting radical scavenging activity against the highly reactive peroxynitrites and hydroxyl radicals [142, 143]. In an *in vitro* system, berberine scavenged ONOO^-^ and its precursors, nitric oxide (NO) and superoxide anion (O^2-^), and increased cell viability. In an *in vivo* lipopolysaccharide plus ischemia-reperfusion system, the administration of *RC* extract resulted in greater inhibition of ONOO^-^, suggesting that berberine could protect against ONOO^-^-induced oxidative damage and that this effect was mainly attributable to the constituent alkaloids. In addition, the free radical scavenging property of berberine can be revealed by its reduction of several free radicals like ABTS (2,2-azinobis(3-ethylbenzothiazoline-6-sul-fonate)), DPPH (2,2-diphenyl1-pi- crylhydrazyl), superoxide, etc. in a concentration dependent pattern [144]. On the other hand, berberine could scavenge both nitric oxide (NO·) and downstream product of RNS, peroxynitrites (ONOO^-^) [142, 143].

The antioxidant activity of berberine has been verified through changes in oxidative stress markers and antioxidant enzymes. As a well-known antioxidant enzyme, superoxide dismutase (SOD) has been shown to be down-regulated by berberine in diabetic mice [3]. In addition to SOD, glutathione (GSH), another antioxidant that often declines during oxidative stress [145] and a substrate of glutathione peroxidase (GSH-Px) in the clearance of peroxides, is also enhanced after berberine application. Moreover, malondialdehyde (MDA), a product of lipid peroxidation that increases during oxidative stress is attenuated by berberine [145, 146]. In addition, berberine relieved oxidative stress in tissues in different organs, including liver, kidney, pancreas and central nervous system, providing multiple targets for the antioxidant effects of berberine in the systemic aging process [137].

In addition to berberine, another component of *RC*, palmatine analog, has been found to penetrate across planar bilayer phospholipid membrane in their cationic forms, and accumulate in isolated mitochondria or in living cultured human cells. Berberine and palmatine inhibit lipid peroxidation in isolated mitochondria at nanomolar concentrations in isolated mitochondria and in living cells. In human cell culture, nanomolar analogs prevented fragmentation of mitochondria and apoptosis induced by exogenous hydrogen peroxide [147]. Pre-incubation of H_2_O_2_ exposed heochromocytoma line PC12 with jatrorrhizine markedly elevated cell viability and activities of antioxidant enzyme (SOD and HO-1), prevented LDH release and MDA production, restored MMP and scavenged ROS formation, suggesting that jatrorrhizine holds potential for neuroprotective effects against H_2_O_2_-induced injury. Jatrorrhizine has been reported to protect against okadaic acid-induced oxidative toxicity by inhibiting the mitogen-activated protein kinases pathways in HT22 hippocampal neurons [148]. Yokozawa *et al.* reported that coptisine, palmatine, magnoflorine, and epiberberine might contribute to the protective effects of* RC* on oxidative stress through inhibition of cellular peroxynitrite generation [149].

This perpetuating oxidative cycle has been proposed to induce the damage of biomolecules thereby disturbing cellular function and leading to multiple aging-associated diseases. Mutiple animal models and clinical studies have established the antioxidant actions of *RC* and its components in various aging-related disorders ranging from diabetes, hyperlipidemia and aging-induced inflammatory response, etc [16]. For instance, the anti-diabetic effect of berberine has been suggested given its ability to lower serum MDA and increase SOD and GSH-px levels in alloxan induced diabetic rats [150]. In STZ-induced diabetic rats and cyclo-phosphamide-induced hepatotoxic rats, berberine could restore the levels of both SOD and GPX, and subsequently reduce elevated levels of lipid peroxidation [3]. Moreover, berberine can inhibit lipid peroxidation, especially LDL oxidation [151]. In addition, berberine can also bind to catalyzing metal ions, which can reduce the concentration of metal ions in lipid peroxidation. Finally, the neuroprotective effect of berberine against chronic brain injury induced by aluminum trichloride in rats has been shown via restoration of neuronal SOD levels and attenuation of MDA contents, which in turn mitigates hippocampal injury and cognitive dysfunction [152]. Jatrorrhizine has been found to protect against okadaic acid-induced oxidative toxicity and apoptosis by inhibiting the mitogen-activated protein kinases pathways in HT22 hippocampal neurons [148].

## 6 Modulation of apoptosis

Apoptosis is required for normal cell turnover and tissue homeostasis. The intrinsic apoptosis pathway is promoted by cellular stresses including DNA damage, activated oncogenes, hypoxia, oxidative stress and irradiation [12]. These stimuli shift the balance of cytoplasmic activities to favor pro-apoptotic factors, Bad and Bax. These factors form a pore structure in the mitochondrial membrane that leads tomitochondrial membrane permeabilization and release of mitochondrial pro-apoptotic factors into the cytoplasm [153], thereby promoting DNA fragmentation and nuclear condensation. The anti-apoptotic “Bcl-2 family” proteins (e.g., Bcl-2, Bcl-x and Bcl-xL) can bind to the pro-apoptotic proteins to inhibit pore formation.

Irregulation of apoptosis has been increasingly implicated in aging and aging-related diseases. Aging-associated disruptions in systemic and inter-cell signaling, combined with cell-autonomous damage and mitochondrial malfunction, result in increased apoptosis in certain cell types, and decreased apoptosis in highly-mitotic cell types [154-156]. Increased apoptosis during aging is implicated in immune system decline, skeletal muscle wasting (sarcopenia), loss of cardiaccells, and neurodegenerative disease. In contrast, cancer cells and senescent cells are resistant to apoptosis, enabling them to increase in abundance during aging. Human serum shows a reduction in apoptotic markers during normal aging [157]. For instance, gastronemius muscle in aging rat showed increased levels of Bax, decreased Bcl-2, activation of caspase-3 and DNA fragmentation [158].

Berberine activates Nrf2 nuclear translocation and inhibits apoptosis induced by high glucose in renal tubular epithelial cells through a phosphatidylinositol 3-kinase/Akt-dependent mechanism [159]. Berberine also significantly attenuates apoptotic death, enhances autophagy and activates the AMPK/mTOR pathway in the hippocampus of diabetic mice and high glucose-treated SH-SY5Y cells. Cells pre-treated with an autophagy inhibitor abolished berberine-inhibited apoptosis. Cells pretreated with an AMPK inhibitor also blocked berberine-inhibited apoptosis and berberine-induced cell autophagy, indicating that berberine inhibits apoptosis via an AMPK-dependent pathway and autophagy process [160]. Such evidence suggests that berberine could protect neuron and renal epithelial cells from apoptosis in the diabetic model, while its apoptotic effects in anti-aging need further investigation.

On the other hand, berberine has also been reported to show pro-apoptotic effect on some human cancer cells. As summarized by Tillhon *et al.,* berberine can alter mitochondrial membrane potential, resulting in a decrease in the expression of Bcl-2 and Bcl-xL, activating caspases and inducing poly (ADP-ribose) polymerase-1 (PARP-1) cleavage. In addition, it has been demonstrated that berberine accumulate on mitochondrial membrane, and subsequently cause the depolarization and fragmentation which may contribute to mitochondrial respiration inhibition. Mitochondrial malfunction subsequently stimulated the release of cytochrome c promoting the formation of ROS that is cytotoxic for cancer cells, and triggers the apoptotic response. It has been shown that berberine triggers the apoptotic process through the formation of ROS, p53 and p21 over-expression and the inhibition of Bcl-2 expression on breast cancer MCF-7 cells [161]. In hepatoma cells, the inhibition of the activity of ERK and PI3K-AKT pathways has been observed. In fact, combined treatment with inhibitors of ERK and AKT and berberine led to a reduction of the expression of MMP-9 and blocking of the invasion process [162]. Apoptosis was induced in human colon carcinoma SW620 cells treated with berberine through the alteration of the JNK/p38 redox/ROS pathways and modulation of ATF3 and NAG-1 proapoptotic factors [163]. The aforementioned evidence shows the dual apoptotic effect of berberine in different aging-related diseases, diabetes and cancer. The role of berberine in the normal aging process, whether it regulates apoptosis in a tissue-specific manner or environment dependent manner is still unknown and requires more investigation.

## Conclusion

**Figure 3.**
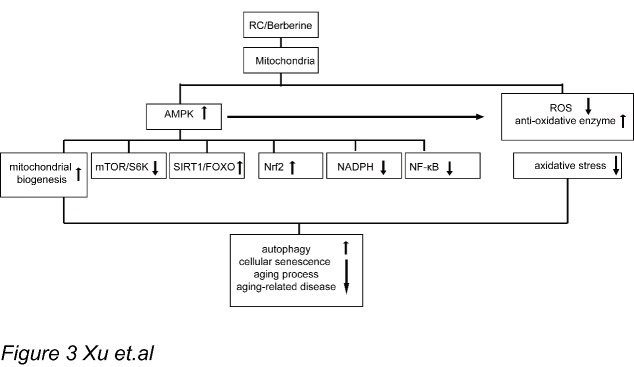
Schematic illustration of the molecular mechanisms and pathways of *RC*/berberine anti-aging and aging-related diseases (1) *RC*/berberine could activate AMPK signaling pathway in the cellular mitochondria. (2) The activation of AMPK by *RC*/berberine inhibits the oxidative stress in mitochondria via down-regulating ROS production and up-regulating anti-oxidative enzymes. (3) The downstream targets of the AMPK signaling regulated by *RC*/berberine include the activation of mitochondrial biogenesis, SIRT1/FOXO and Nrf2 signaling, and inhibition mTOR/S6K, NADPH and NF-??B pathways. (4) Via regulating the downstream productions of the signaling pathways mentioned above, *RC*/berberine could activate cellular autophagy which finally inhibits cellular senescence, aging process and the pathological process of aging-related diseases.

In summary, Chinese herb rhizoma coptidis and its main bioactive components has been used in aging-related diseases widely, i.e. hyperglycemia, hyperlipidemia and some neurodegenerative diseases. In particular, the main component, berberine, has found to extend life span, overall health, and attenuate premature cellular senescence in animal models. The anti-aging property of *RC* is evidenced by changes in associated markers of oxidative stress, apoptosis and inflammatory cytokines after berberine administration. The mechanism of these activities as shown in Fig. 3 involve multiple cellular kinase and signaling pathways include anti-oxidation, activation of AMPK signaling and its downstream targets, including mTOR/S6, Sirtuin 1/forkhead box transcription factor O3 (FOXO3), nuclear factor erythroid-2 related factor-2 (Nrf2), nicotinamide adenine dinucleotide (NAD^+^) and nuclear factor-κB (NF-κB) pathways. Given the increased interest in berberine as a natural supplement that can potentially combat aging, the molecular processes of berberine’s antioxidant and anti-inflammatory properties merit further investigation. In addition, more research is needed to improve the bioavailability of berberine.
